# A patient with Melorheostosis manifesting with features similar to tricho-dento-osseous syndrome: a case report

**DOI:** 10.1186/1752-1947-2-51

**Published:** 2008-02-19

**Authors:** Ali Al Kaissi, Martin Skoumal, Katharina Roetzer, Franz Grill, Klaus Klaushofer

**Affiliations:** 1 Ludwig Boltzmann Institute of Osteology at the Hanusch Hospital of WGKK and AUVA Trauma Centre Meidling, 4th Medical Department, Hanusch Hospital, Heinrich Collin Str.A-30 Vienna, Austria; 2 Institute for Rheumatology of the Kurstadt Baden in Cooperation with the Danube – University Krems, A-2500 Baden, Austria; 3 Orthopaedic Hospital of Speising, Paediatric Department, Speisinger Str. 109, Vienna, Austria

## Abstract

**Introduction:**

A case of melorheostosis in association with tricho-dento-osseous (TDO) syndrome has been encountered.

**Case presentation:**

The clinical and the radiographic manifestations of melorheostosis have been encountered in a 41-year-old man. Mutations in the 13 exons and flanking intronic regions of the LEMD3-gene have not been detected. His phenotypic features were consistent but not completely diagnostic for tricho-dento-osseous syndrome (TDO). We report what might be a novel syndromic association.

**Conclusion:**

Melorheostosis has not previously been reported to be a part of TDO and an extensive review of the literature suggests that the constellation of hair, tooth and bone abnormalities found in our patient either represents an unusual variant of tricho-dento-osseous syndrome or a new syndrome.

## Introduction

Melorheostosis is a rare disorder, also known as Leri disease or flowing periosteal hyperostosis [1]. It is a form of sclerosing bone dysplasia characterized radiologically, by the appearance in the long bones of "melting wax flowing down a candle". This appearance is the result of longitudinal bars of hyperostosis along single or contiguous bones. Melorheostosis generally manifests during early childhood, although occasionally the initial signs appear in adulthood [1]. It may occur in association with other types of sclerosing bone dysplasias such as osteopoikilosis and osteopathia striata [2], and with tumour-forming disease such as tuberous sclerosis and neurofibromatosis [3]. Vascular lesions associated with melorheostosis include haemangiomas, vascular nevi, varices, glomus tumours, arteriovenous malformations and aneurysms [4].

Classically, melorheostosis affects the long bones of the skeleton, especially those in the lower extremities. Involvement of the axial skeleton is rare [5]. We describe a male with a unique constellation of abnormalities consisting of a severe form of melorheostosis of the right upper limb, curly hair, anodontia, and marked sclerosis of the skull base that extended to involve the upper cervical spine. Other long bone sclerosis was noticed, but it was of less intensity. Given the involvement of the hair and teeth, the overall clinical and radiographic features in our patient resembled tricho-dento-osseous syndrome with additional features.

## Case presentation

A 41-year-old man presented with a 26-year history of melorheostosis affecting the right forearm and hand. His right middle finger was operated on at the age of 15-years, but recently, the pain had increased in intensity and the contractures had worsened. The second finger of his right hand had become deformed. He underwent proximal interphalangeal arthrodesis to ease the pain and correct the deformity. He had a history of gradual loss of teeth from an early age, and by the age of 20 years, he had lost all his teeth. Seven years later he developed sensorineural deafness of the right ear. A fracture of the distal part of the right humerus occurred following moderate trauma. The family history was non-contributory.

**Figure 1:**
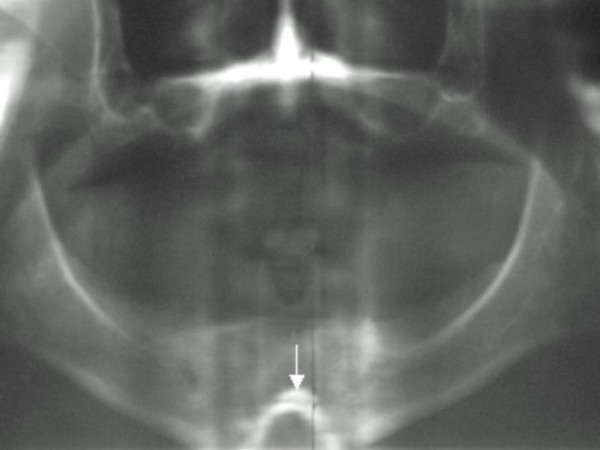
Radiographic pantogram showed total anodontia, in addition there was inverted V shaped dysplastic mental protuberance (arrow).

Examination revealed short stature (-2SD), curly/kinky hair and frontal bossing. Oral examination showed anodontia (fig 1) and there was dimpling over his chin. He was deaf on the right side with normal hearing on the left side. He had an upper thoracic kyphosis, and a unilateral deformity of the right forearm and hand. This region was firm and painful to palpation, but the overlying skin showed no evidence of discoloration, thickening, or any local increase in temperature. Bilateral bowing of the forearms and the legs was noted. A 4-inch hyperpigmented patch was seen on the left side of his trunk superimposed by freckling spots, but there was no other freckling in the axillae or elsewhere. Ophthalmological, and cardiological examinations were normal, as was his intelligence.

**Figure 2:**
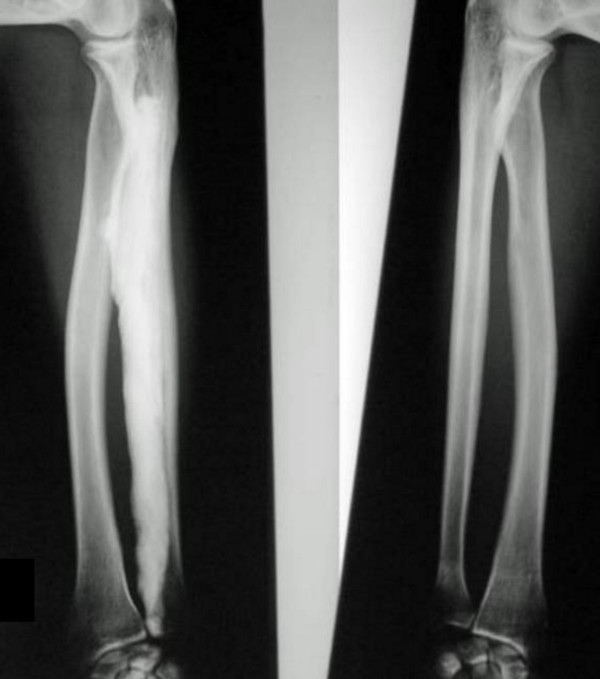
**Radiograph of the forearms showed, typical cortical thickening, mostly along the right radius, massive extensive thickening and sclerosis extends along the carpal bones respectively.** There was mild sclerosis of the ulnae with bowing; similarly the radius on the left side is also mildly sclerotic.

**Figure 3:**
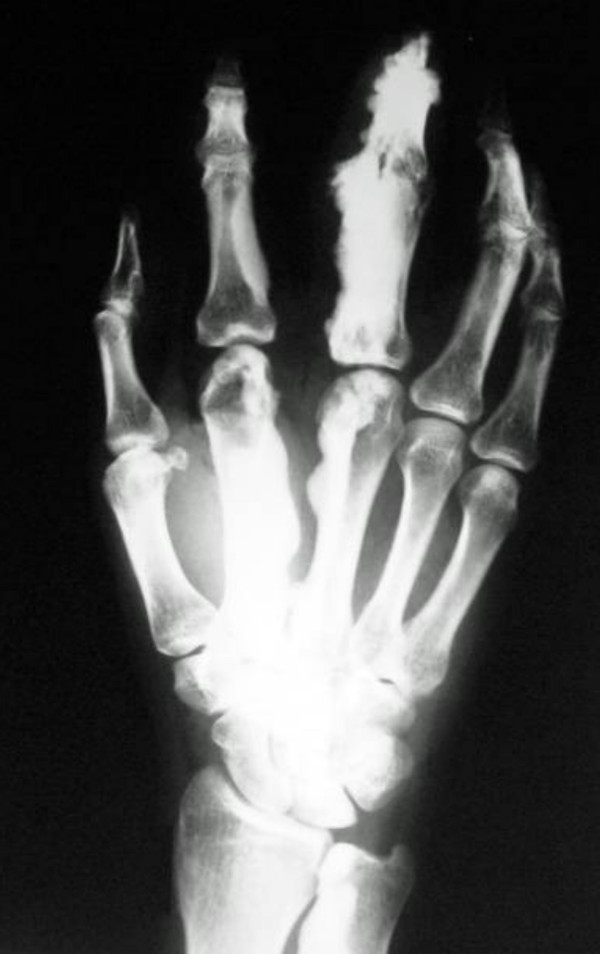
**There is massive sclerosis over the second and third digits (carpo-metacarpo-phalangeal)**. The sclerosis extends to involve the distal and middle phalanges of 4th digit as well as the carpal bones.

Radiographic examinations are shown in figures 1, 2, 3

Blood biochemistry revealed moderate hypercholestrolaemia, and the triglycerides showed mild elevated levels. Serum calcium, phosphorus, and alkaline phosphatase levels were normal. Endocrinological examination of the thyroid hormones, PTH, and adrenocorticotropic hormones showed no abnormalities. Our patient underwent a genetic investigation of the LEMD3-gene; Genomic DNA was extracted from the whole blood samples and polymerase chain reaction (PCR) was performed to amplify the 13 exons and flanking intronic regions of the LEMD3-Gene (primer sequences are available on request). The PCR products were analysed by direct sequencing (MWG Biotech G, Ebersberg, Germany). We were unable to detect any mutations in the 13 exons and flanking intronic regions of the LEMD3-gene in our patient (NCBI reference sequence: NC-000012).

Radiographic examination:

## Discussion

Melorheostosis is a rare hyperostotic, benign, sclerosing bone dysplasia [1,2]. It may present in a monostotic (involvement of one bone), polyostotic, or monomelic (involvement of one limb) form. The aetiology is controversial rather than definite. It was suggested that neural infection (analogous to herpes zoster) could result in lesions spread along the distribution of the associated sensory nerve root, with resultant scarring and hyperostosis [5]. Other suggestions of aetiology such as a vascular disorder, a degenerative lesion of connective tissue or embryonic damage have also been proposed [6]. Most interestingly, it has been thought to occur as a result of an embryonal metameric disturbance, which causes a failure in intramembraneous, and to a lesser extent, endochondral ossification [1]. One of the typical target sites of endochondral ossification is the skull base. In our patient, however, the persistence of subdental synchondrosis was either secondary to defective ossification of the first spinal sclerotome and or/it was secondary to the development of an asymptomatic non-union type II dens fracture [7].

Patients with melorheostosis usually present with pain and limitation of joint movement. The overlying skin may be tense, shiny, erythematous or hyperaemic. Muscle atrophy and linear scleroderma may be other features. Roger et al [3] reported a patient with features of melorheostosis associated with minimal change nephrotic syndrome, mesenteric fibromatosis and multiple capillary haemangiomas of the trunk. However, no hair and/or dental involvement was reported. Zeiller et al [8] reported the development of a severe myelopathy of the cervicothoracic spine associated with melorheostosis. Osteosarcoma and desmoid tumour were described in two separate reports in patients with melorheostosis [5].

Hellemans et al [9] have previously identified the gene locus of melorheostosis. It was found to be allelic with osteopoikilosis and Buschke-Ollendorff syndrome. They thought in might be allelic with melorheostosis [10]. Our patient, however, manifested melorheostosis as a symptom complex. The overall malformation complex was not relevant with previous reports [9,10].

Differentiating melorheostosis from other sclerosing bone dysplasias is mandatory. Progressive diaphyseal dysplasia (Camurati-Engelmann syndrome) is characterised by a mixed sclerosing bone dysplasia affecting predominantly intramembraneous ossification that is inherited in an autosomal dominant fashion. Patients usually have symmetrical involvement of the extremities. Pathologically there is progressive bone formation along both the periosteal and endosteal surfaces of the long tubular bones. Radiologically there is sclerosis and thickening of the cortex of the skull and long bones, involving both the diaphyses and the metaphyses, but not the epiphyses [11]. Weismann-Netter-Stuhl syndrome is another sclerosing bone dysplasia (toxopachyosteose diaphysaire tibio-peroniere) also manifesting with progressive bone sclerosis, predominately involving the diaphyses and not the whole bone components [12]. Neither of these conditions fit the patient reported here.

Osteopoikilosis is another clinical entity of bone sclerosis in which the radiological lesions appear as small areas of bony sclerosis throughout the skeleton, although largely sparing the skull. The skin lesions may appear at any time from birth to adult life and consist of pea-sized papules of Buschke-Ollendorff syndrome [13]. Osteopathia striata is the combination of vertical striations of the metaphyses of the long bones, a large head with sclerosis, thickening of the skull vault and a variety of other manifestations including cleft palate, mental retardation, and sensorineural deafness or other signs of cranial nerve compression including facial paralysis [2]. All of these conditions were considered, but the overall clinical and radiographic features do not fit our patient.

Tricho-dento-osseous syndrome (TDO) was probably first described by Robinson and Miller [14]. The main features are enamel hypoplasia of the teeth, mild osteosclerosis (particularly of the skull), brittle nails and blond, dry, curly hair. All affected individuals have taurodontism, although with variable expression. 85% of affected individuals have kinky or curly hair. Ninety-seven percent of patients with TDO have thickened cranial bones without pneumatisation of the sinuses [13-15]. Melorheostosis has not been described in connection with TDO.

## Abbreviations

TDO: Tricho-Dento-Osseous

## Competing interests

The author(s) declare that they have no competing interests.

## Authors' contributions

All authors read and approved the final manuscript.

## Consent

Written informed consent was obtained from the patient for publication of this case report and accompanying images. A copy of the written consent is available for review by the Editor-in-Chief of this journal.
